# Exploring the interplay of cognitive flexibility, self-regulation, and perceived control in enhancing psychological recovery among competitive athletes

**DOI:** 10.3389/fpsyg.2026.1789097

**Published:** 2026-04-01

**Authors:** Dawei Yu, Nan Pei

**Affiliations:** 1 College of Teacher Education (Physical Education), Taizhou University, Taizhou, Zhejiang, China; 2 Physical Education Department, Changchun University of Chinese Medicine, Changchun, Jilin, China

**Keywords:** cognitive flexibility, competitive athletes, perceived control, PLS-SEM, psychological recovery, self-regulation

## Abstract

**Introduction:**

Psychological recovery is essential for maintaining performance and well-being among competitive athletes, yet the cognitive and self-regulatory mechanisms underlying recovery remain insufficiently understood. From a resource-based perspective, this study investigates how cognitive flexibility contributes to psychological recovery through self-regulation and examines the moderating role of perceived control in this process.

**Methods:**

Data were collected through face-to-face surveys from 467 competitive athletes in China. A moderated mediation model was tested using partial least squares structural equation modeling (PLS-SEM) to examine the relationships among cognitive flexibility, self-regulation, perceived control, and psychological recovery.

**Results:**

The results indicate that cognitive flexibility is positively associated with self-regulation, which in turn positively predicts psychological recovery, supporting a significant indirect effect through self-regulation. Furthermore, perceived control significantly moderates the indirect relationship between cognitive flexibility and psychological recovery. Specifically, the mediated effect weakens as perceived control increases, indicating a boundary condition in the recovery process.

**Discussion:**

This study advances recovery research by integrating cognitive adaptability, regulatory processes, and contextual perceptions. The findings conceptualize psychological recovery as a cognitively driven and self-regulated process. Practically, the results suggest that coaches and sport psychologists can promote athletes’ recovery by fostering cognitive flexibility and structured self-regulation strategies while carefully managing perceptions of control to avoid counterproductive overconfidence or rigid self-management.

## 1 Introduction

Psychological recovery has become a central concern in contemporary sport psychology as competitive athletes are increasingly exposed to intensified training loads, congested competition schedules, and heightened performance expectations (Beckmann, 2023). Sustained exposure to such demands places athletes at risk of psychological strain, impaired wellbeing, and performance decrements if adequate recovery processes are not achieved. Accordingly, recovery is now widely recognized as a critical component of long-term performance sustainability rather than a peripheral or secondary outcome of training cycles. In sport psychology, recovery is generally conceptualized as a process through which psychophysiological resources depleted during training and competition are restored, enabling athletes to return to baseline functioning and readiness for subsequent performance demands (Kellmann et al., 2018; Balk et al., 2019). Within this broader framework, the present study focuses specifically on psychological recovery, defined as the restoration of cognitive, emotional, and motivational resources following sport-related training demands, rather than recovery from injury, illness, or concussion (Balk and Englert, 2020; Eccles and Kazmier, 2019). This distinction clarifies that the study examines the recovery of psychological capacity itself, rather than the broader psychology of recovery across physical and medical domains (Eccles et al., 2022). Despite this growing recognition, much of the existing literature has focused on physiological aspects of recovery, with comparatively less attention devoted to the psychological mechanisms that enable athletes to recover effectively from competitive stressors.

Emerging perspectives suggest that psychological recovery is not a passive consequence of rest but an active process shaped by how athletes cognitively appraise demands and regulate their internal states following performance. Recent research on mental detachment, mental rest, and post-exertion cognitive processes emphasizes that athletes’ appraisal patterns and post-training thought processes critically influence the effectiveness of recovery (Balk et al., 2021; Wilson and Young, 2023). In this regard, adaptive cognition may help athletes disengage from maladaptive ruminative patterns, reframe performance experiences, and flexibly adjust to situational changes, all of which are essential for effective psychological restoration. However, while such cognitive capacities have been examined in relation to coping and performance, their role in post-competition recovery remains theoretically underdeveloped.

At the same time, self-regulatory processes have been identified as a cornerstone of effective functioning in high-performance environments (Beckmann et al., 2023). Research has consistently shown that athletes who are able to regulate their goals, emotions, and behaviors more effectively tend to cope better with competitive demands and sustain higher levels of functioning over time (Popovych et al., 2022; Trotter et al., 2021; Vasilopoulos and Ellefson, 2021). Conceptualizations of recovery self-regulation suggest that athletes must intentionally manage attention, arousal, and emotional states to facilitate effective restoration following exertion (Balk and Englert, 2020). Nevertheless, existing studies have often treated self-regulation as an outcome or a general skill, rather than explicitly positioning it as a mechanism through which cognitive capacities translate into recovery-related benefits.

Moreover, accumulating evidence indicates that psychological processes in sport rarely operate uniformly across individuals or contexts. Athletes’ perceptions of their ability to influence outcomes and manage situational demands appear to shape how psychological resources are deployed and with what effectiveness. Perceived control, in particular, has been associated with more adaptive stress responses, better coping, and improved wellbeing in sport and performance settings (Parker et al., 2023). However, perceived control has rarely been integrated into theoretical models explaining recovery from training demands, leaving unclear how contextual appraisals shape the effectiveness of cognitive and self-regulatory mechanisms. As a result, the conditions under which self-regulation most effectively supports psychological recovery remain insufficiently specified.

In response to these gaps, the present study adopts an integrative perspective to examine psychological recovery as a cognitively driven and contextually contingent process among competitive athletes. By simultaneously considering adaptive cognitive capacity, self-regulatory mechanisms, and perceived control within a moderated mediation framework, the study seeks to advance understanding of how recovery is associated with cognitive and regulatory processes. In doing so, the study responds to recent calls for more nuanced, theory-driven models that move beyond simple direct effects and capture the dynamic interplay between cognitive resources, regulatory processes, and contextual appraisals in sport psychology research (Hobfoll, 1989; Kellmann et al., 2018). This approach not only extends existing recovery literature but also offers a more comprehensive foundation for understanding sustainable psychological functioning in competitive sport contexts.

## 2 Theoretical background

The present study is theoretically grounded in Conservation of Resources (COR) theory, originally proposed by Hobfoll (1989), which offers a comprehensive framework for understanding how individuals respond to stress, recovery, and performance demands. COR theory posits that individuals are motivated to obtain, retain, and protect valued resources, including personal characteristics, cognitive capacities, emotional energies, and contextual conditions, and that psychological strain arises when these resources are threatened or depleted (Hobfoll, 1989). Subsequent extensions of the theory have emphasized that resources rarely operate in isolation but instead form dynamic resource caravans that shape individuals’ capacity to cope with ongoing demands across time and contexts (Hobfoll, 2001; Hobfoll et al., 2018). COR theory has been extensively applied across diverse domains, including occupational stress, health psychology, education, and sport, consistently demonstrating that individuals who possess greater personal and contextual resources are better able to manage stress, recover from strain, and sustain long-term functioning (Halbesleben et al., 2014; Hobfoll et al., 2018). Within sport psychology, recovery from training demands can be conceptualized as a resource restoration process in which depleted cognitive and emotional resources must be replenished to prevent underrecovery and performance decline (Kellmann et al., 2018; Balk et al., 2019).

**Figure 1:**
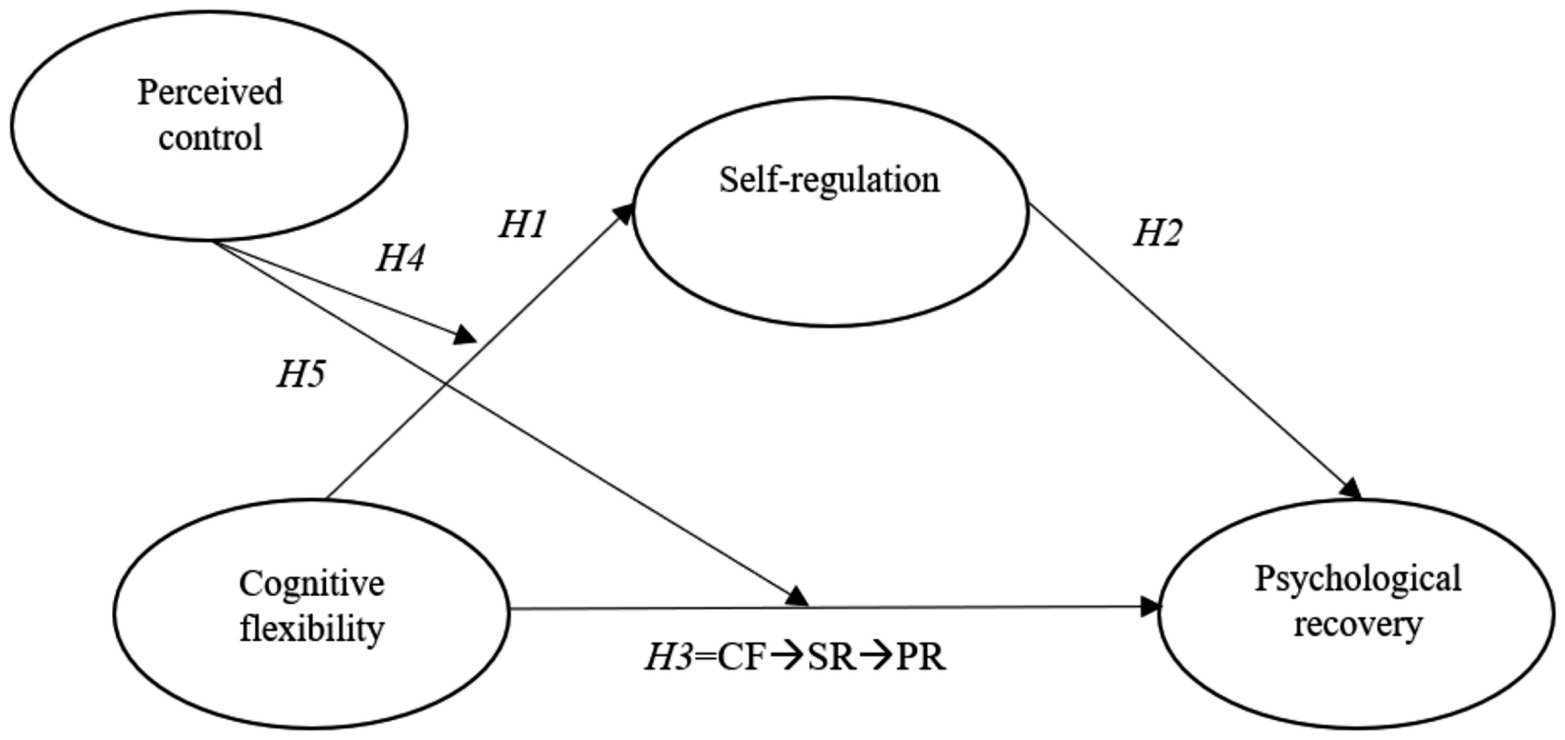
Conceptual model.

Within competitive sport contexts, COR theory provides a particularly relevant lens for conceptualizing psychological recovery as a resource-dependent process rather than a passive consequence of rest. Specifically, psychological recovery from training refers to the restoration of cognitive, emotional, and motivational capacity following exertion, enabling athletes to re-engage effectively with subsequent performance demands (Balk and Englert, 2020; Eccles and Kazmier, 2019). From this perspective, cognitive flexibility can be understood as a critical personal cognitive resource that enables athletes to adaptively appraise competitive demands, reframe setbacks, and avoid rigid, resource-depleting thought patterns (Hobfoll, 1989; Balk and Englert, 2020). Self-regulation, in turn, represents a higher-order regulatory resource that is associated with the management of emotions, attention, and recovery-related behaviors (Balk and Englert, 2020). Consistent with recent research on recovery self-regulation, regulatory efforts such as detachment, emotional control, and attentional shifting are mechanisms through which athletes actively restore depleted psychological resources after training (Balk et al., 2021; Wilson and Young, 2024). Moreover, perceived control functions as a contextual and appraisal-based resource that conditions the effectiveness of resource investment, such that regulatory efforts are more likely to result in psychological recovery when athletes believe they can influence outcomes and manage situational demands (Hobfoll, 2001; Halbesleben et al., 2014). In sport contexts, perceived control has been linked to adaptive stress appraisal and effective coping, suggesting that it may strengthen or weaken the translation of cognitive and regulatory resources into recovery outcomes (Eccles et al., 2022). By integrating cognitive flexibility, self-regulation, and perceived control within a moderated mediation framework (Figure 1), the present study extends COR theory into the domain of athletic recovery by explicating how cognitive resources fuel regulatory processes and how contextual resources shape the strength of resource-to-recovery pathways, thereby advancing theoretical understanding of recovery in high-demand performance environments.

### 2.1 Perceived control in sports contexts

Perceived control has been consistently identified as a central appraisal construct in sport psychology, influencing how athletes interpret demands, allocate effort, and regulate responses under pressure. In performance settings, perceived control refers to athletes’ beliefs regarding their capacity to influence outcomes, manage situational demands, and exert agency over stressors encountered during training and competition (Eccles and Kazmier, 2019). Research demonstrates that higher perceived control is associated with adaptive stress appraisal, increased motivational persistence, and more effective coping strategies in elite and non-elite athletes (Lavoie et al., 2021; Wilson and Young, 2023). Conversely, diminished control appraisals have been linked to disengagement, rumination, and impaired psychological restoration following exertion (Eccles et al., 2022).

Within recovery research, perceived control is particularly relevant because recovery from training demands requires active engagement in regulatory behaviors, such as emotional detachment, attentional shifting, and arousal modulation (Balk and Englert, 2020). Athletes who believe they can influence their performance context are more likely to invest in such regulatory efforts, whereas low perceived control may undermine the perceived utility of recovery strategies. From a resource-based perspective, perceived control functions as a contextual resource that shapes the effectiveness of personal resources, thereby conditioning the extent to which self-regulation translates into psychological recovery (Hobfoll, 2001; Hobfoll et al., 2018).

## 3 Hypotheses

### 3.1 Relationship between cognitive flexibility and self-regulation

Adaptive cognitive functioning is widely regarded as a prerequisite for effective self-management in high-pressure environments. Gholami et al. (2022) note that individuals with greater cognitive flexibility are better able to disengage from ineffective thought patterns and adjust strategies when situational demands change. In sport settings, flexible cognitive processing has been associated with enhanced coping capacity and reduced stress reactivity, suggesting that athletes who can cognitively adapt are more capable of maintaining regulatory control during performance (Başoğlu et al., 2025). In addition, Logan et al. (2023) report that flexible appraisal styles allow athletes to recalibrate goals and attentional focus more efficiently when confronted with setbacks. Research in executive functioning further reinforces that cognitive flexibility supports the monitoring and adjustment components of self-regulation, particularly when habitual responses are insufficient (Yongtawee et al., 2022). From a motivational standpoint, adaptive cognition enables athletes to persist in regulatory efforts rather than disengaging when faced with obstacles, a pattern observed across elite and non-elite samples (Król and Gruszka, 2023). Within a resource-based framework, cognitive flexibility can be conceptualized as a personal resource that facilitates the effective deployment of self-regulatory resources under stress (Hobfoll, 1989; Halbesleben et al., 2014). Consistent with recovery self-regulation perspectives in sport, adaptive cognitive appraisal enables athletes to shift attention away from stress-inducing stimuli and toward restoration-oriented regulation strategies following training demands (Balk and Englert, 2020). Collectively, these findings suggest that greater cognitive flexibility strengthens athletes’ capacity for self-regulation.

Accordingly, it is hypothesized that:

*H1*: Cognitive flexibility is positively related to self-regulation.

### 3.2 Relationship between self-regulation and psychological recovery

Recovery from competitive stress has increasingly been framed as an active process requiring deliberate regulation rather than mere cessation of activity. For instance, Popovych et al. (2022) demonstrate that athletes with stronger self-regulatory skills are better able to manage performance demands and sustain adaptive functioning over time. Extending this view, Beckmann (2023) argue that recovery itself constitutes a self-regulatory act, involving the intentional downregulation of arousal and cognitive engagement after exertion. Empirical evidence further supports this proposition, showing that effective self-regulation is associated with more favorable recovery–stress profiles and lower emotional exhaustion among athletes (Beckmann et al., 2023; Trotter et al., 2021). In addition, Wilson and Young (2024) provide qualitative evidence suggesting that difficulties in regulating recovery-related thoughts and behaviors may be associated with maladaptive recovery experiences and increased psychological strain. Studies conducted in endurance and team sport contexts further suggest that athletes who actively regulate emotions and attention experience greater psychological restoration following competition (Balk and Englert, 2020; Balk et al., 2019). From a theoretical perspective, self-regulation enables athletes to conserve and replenish depleted psychological resources, thereby supporting recovery in line with conservation of resources principles (Hobfoll, 2001). Within training contexts, such regulatory processes facilitate psychological recovery by enabling athletes to disengage from performance-related rumination and actively restore cognitive and emotional capacity (Eccles and Kazmier, 2019; Balk et al., 2021). Taken together, the literature consistently indicates that self-regulation plays a central role in facilitating psychological recovery.

Thus, it is hypothesized that:

*H2*: Self-regulation is positively related to psychological recovery.

### 3.3 Mediating role of self-regulation between cognitive flexibility and psychological recovery

Although adaptive cognition has been linked to positive sport-related outcomes, the mechanisms through which it contributes to recovery remain insufficiently specified. Prior research suggests that cognitive resources rarely exert their influence directly but instead operate through intermediate self-regulatory processes. Recent sport recovery research emphasizes that recovery outcomes depend on athletes’ ability to regulate post-training cognitions and emotional responses, rather than on cognitive capacity alone (Balk and Englert, 2020; Eccles et al., 2022). Similarly, Ronkainen et al. (2024) show that flexible cognitive appraisals support recovery indirectly by enabling athletes to implement adaptive coping and regulation strategies. Research on executive control indicates that cognitive flexibility supports regulatory monitoring and adaptive adjustment in response to changing demands (Diamond, 2013; Miyake et al., 2000). These executive processes may facilitate the effective management of recovery-related behaviors in sport contexts. In contrast to prior work that has treated cognitive flexibility and recovery as directly linked constructs, the present study proposes that self-regulation functions as the process mechanism through which adaptive cognition is statistically associated with psychological restoration. From a COR perspective, cognitive flexibility serves as an upstream resource that strengthens self-regulation, which in turn facilitates resource restoration following stress exposure (Hobfoll, 1989; Halbesleben et al., 2014). These arguments collectively support the proposition that self-regulation explains how cognitive flexibility contributes to psychological recovery.

Therefore, it is hypothesized that:

*H3*: Self-regulation mediates the relationship between cognitive flexibility and psychological recovery.

### 3.4 Moderating role of perceived control on self-regulation

A review of prior research suggests that psychological mechanisms in sport rarely exert uniform effects across individuals or situations (Trotter et al., 2021; Wilson and Young, 2024; Yongtawee et al., 2022), making it theoretically insufficient to assume that self-regulatory processes operate with equal strength for all athletes. Contemporary stress and performance models emphasize that the effectiveness of psychological skills depends not only on their presence but also on the contextual and appraisal-based conditions under which they are deployed (Fletcher and Sarkar, 2012; Meijen et al., 2020). In this regard, perceived control has been identified as a critical boundary condition that determines whether individuals invest in, sustain, or withdraw regulatory effort when confronted with demanding situations. Gomes et al. (2022) argues that control appraisals fundamentally shape motivational engagement, such that individuals who perceive higher control are more likely to persist in effortful regulation, whereas low perceived control fosters disengagement and helpless responses. Empirical evidence in sport contexts supports this proposition, showing that athletes with higher perceived control exhibit more adaptive coping responses and more favorable recovery–stress balance, particularly during periods of intensified demand (Lavoie et al., 2021). Research on the psychology of rest and recovery further indicates that athletes’ beliefs about their capacity to influence training demands shape the effectiveness of regulatory strategies aimed at restoring mental energy (Eccles et al., 2022; Wilson and Young, 2023). Moreover, even well-developed regulatory skills may fail to translate into effective outcomes when athletes perceive limited control over their environment, as low control undermines expectancy and reduces the perceived utility of regulatory effort (Parker et al., 2023). From the perspective of conservation of resources theory, perceived control represents a contextual resource that conditions the effectiveness of personal resource investment, such that self-regulatory resources yield stronger outcomes when sufficient contextual resources are available (Hobfoll, 2001; Hobfoll et al., 2018). Accordingly, when athletes perceive higher control over training demands, self-regulatory efforts are more likely to translate into effective psychological recovery, whereas under low perceived control, the impact of self-regulation is attenuated. Collectively, these arguments demonstrate that moderation is theoretically necessary to capture variability in how self-regulation influences recovery outcomes across differing levels of perceived control.

Thus, it is hypothesized that:

*H4*: Perceived control moderates the relationship between self-regulation and psychological recovery, such that the positive relationship is expected to be stronger when perceived control is low and weaker when perceived control is high.

### 3.5 Moderated mediating role of perceived control on psychological recovery

While moderation captures whether the strength of a single relationship varies across levels of perceived control, it does not fully explain how contextual appraisals shape the operation of underlying psychological mechanisms. Increasingly, scholars argue that psychological processes in performance contexts are best understood as conditional chains of influence, in which indirect effects themselves vary depending on situational or appraisal-based conditions (Hayes, 2018). In the context of recovery, it is therefore insufficient to assume that the mediating role of self-regulation between cognitive flexibility and psychological recovery operates uniformly across athletes. Rather, the effectiveness of this indirect pathway is likely contingent on athletes’ perceived control, which determines whether regulatory efforts translate into meaningful recovery gains. From a conservation of resources perspective, resource investment processes are most effective when personal resources and contextual resources are jointly available, whereas misalignment between these resources weakens gain spirals and limits recovery outcomes (Hobfoll, 2001; Hobfoll et al., 2018).

Empirical evidence supports this conditional logic, showing that regulatory skills yield stronger wellbeing and recovery benefits under conditions of higher perceived control, while their impact is attenuated when control appraisals are low (Popovych et al., 2022; Vasilopoulos and Ellefson, 2021). Moreover, recent sport psychology research highlights that recovery is a dynamic self-regulatory process whose effectiveness depends on contextual affordances and athletes’ appraisals of influence over their environment, underscoring the need to move beyond simple mediation models (Balk and Englert, 2020; Beckmann, 2023; Beckmann et al., 2023). Methodologically, moderated mediation models provide a more accurate representation of such conditional processes by explicitly testing whether the indirect effect of cognitive flexibility on psychological recovery through self-regulation varies across levels of perceived control (Hayes, 2018). Accordingly, examining moderated mediation allows the present study to capture not only how recovery is facilitated but also when and under what conditions this facilitation is most effective.

Thus, it is hypothesized that:

*H5*: Perceived control moderates the indirect relationship between cognitive flexibility and psychological recovery via self-regulation, such that the indirect association is stronger when perceived control is low and weaker when perceived control is high.

## 4 Methodology

### 4.1 Research design and data collection procedure

This study employed a cross-sectional quantitative research design to examine the relationships among cognitive flexibility, self-regulation, perceived control, and psychological recovery among competitive athletes. The cross-sectional design was selected to test theoretically grounded associations among personal and contextual resources within a single assessment context, rather than to establish temporal or causal effects. Accordingly, all mediation findings should be interpreted as statistical indirect associations rather than evidence of temporal causality. Data were collected using a face-to-face questionnaire survey conducted in China, which was chosen to enhance response accuracy, reduce item non-response, and ensure that participants clearly understood the survey items. Face-to-face administration is particularly appropriate in athletic settings, where direct access to participants during training sessions or competitions allows for controlled data collection and improved data quality (Bowling, 2005; Denscombe, 2017). Questionnaires were administered in controlled environments immediately following scheduled training sessions, rather than during competition or high-stress periods, to reduce situational variability in recovery states. Trained research assistants administered the questionnaires on site and remained available to clarify procedural questions without influencing respondents’ answers. Prior to participation, athletes were informed about the academic purpose of the study, assured that participation was voluntary, and guaranteed anonymity and confidentiality. Informed consent was obtained from all respondents in accordance with standard ethical research practices.

### 4.2 Sample and sampling procedure

The target population consisted of competitive athletes actively engaged in organized sports training and competition. A purposive sampling approach was used to recruit athletes from sports academies, universities, and professional training centers across multiple regions in China. A total of 550 questionnaires were distributed in person. After excluding incomplete responses and questionnaires with patterned or inconsistent answers, 467 valid questionnaires were retained for analysis, yielding a valid response rate of approximately 85%. This sample size exceeds the minimum requirements for PLS-SEM analysis (Hair et al., 2021), particularly for models incorporating mediation and moderation effects, and provides sufficient statistical power to detect meaningful relationships among constructs.

To enhance clarity regarding representativeness, athletes were categorized according to competitive level. Approximately 28.4% competed primarily at university level, 46.7% at provincial level, and 24.9% at national-level competitions. This distribution indicates that the sample included athletes exposed to structured, high-performance training environments across multiple competitive tiers.

To contextualize the competitive level of the sample using established international classification frameworks, we mapped participants onto the performance-tier criteria proposed by Swann et al. (2015) and the participant classification framework developed by McKay et al. (2022). According to these frameworks, athletes can be categorized based on competition level, training structure, and performance standard. The present sample consisted primarily of academy-level and university-level competitive athletes who regularly engaged in structured training programs and participated in regional or national competitions. Based on McKay et al.’s (2022) taxonomy, the majority of participants would correspond to Tier 3 (highly trained/national-level) or Tier 2 (trained/developmental competitive) athletes rather than world-class elite performers.

Within the Chinese sport system, academy athletes typically follow regimented training schedules comparable to structured development pathways in other countries, while university athletes often compete in intercollegiate leagues and national student competitions. This classification provides international readers with clearer context regarding the performance caliber and training demands of the sample.

### 4.3 Demographic characteristics

Demographic information was collected to describe the sample and control for potential confounding effects. The final sample of 467 athletes consisted of approximately 58.2% males and 41.8% females. In terms of age, 32.5% of respondents were aged 18–22 years, 41.1% were aged 23–27 years, 18.6% were aged 28–32 years, and 7.8% were above 32 years. Regarding competitive experience, 29.3% reported less than 3 years of experience, 38.5% reported 3–6 years, 21.8% reported 7–10 years, and 10.4% reported more than 10 years of competitive experience. Athletes represented a range of individual and team sports, including athletics, basketball, football, badminton, volleyball, and martial arts.

## 5 Measures

All constructs were measured using established self-report scales adapted to the competitive sports context (see Appendix A). Responses were recorded on a five-point Likert scale ranging from 1 (strongly disagree) to 5 (strongly agree). All items were framed to reflect athletes’ experiences during the past week to ensure temporal consistency across constructs and to reduce discrepancies between dispositional and state-oriented measures.

All instruments were translated and contextually adapted for use in Chinese sport settings using a back-translation procedure. Two bilingual experts independently translated the items into Chinese, and discrepancies were resolved through discussion. A pilot test was conducted with 35 competitive athletes to assess clarity, contextual relevance, and item comprehension. Minor wording adjustments were made to ensure alignment with sport-specific terminology. Preliminary reliability analyses from the pilot sample indicated acceptable internal consistency across constructs (Cronbach’s *α* > 0.80).

Cognitive flexibility was assessed using a six-item scale adapted from the Cognitive Flexibility Inventory, which captures individuals’ ability to shift perspectives and adapt to changing situational demands (Dennis and Vander Wal, 2010). Although cognitive flexibility is often conceptualized as a relatively stable personal resource, in the present study it was assessed using a recent-week framing to capture contextually activated cognitive adaptability within training environments. Self-regulation was measured with eight items drawn from prior work on self-regulatory processes in performance contexts, focusing on goal management, emotional control, and behavioral regulation (Zimmerman, 2000; Toering et al., 2009). Specifically, items reflected planning, emotional regulation, and attentional control dimensions of self-regulation. These items were selected to represent regulatory processes relevant to training engagement and post-exertion recovery management. Perceived control was measured using a five-item scale adapted from established perceived control and mastery measures, assessing athletes’ beliefs about their ability to influence outcomes and manage competitive challenges (Skinner, 1996; Lachman and Weaver, 1998). The scale captures appraisal-based beliefs regarding agency within recent sport-related situations. Finally, psychological recovery was assessed with six items adapted from recovery and detachment measures commonly used in stress and sport psychology research (Sonnentag and Fritz, 2007; Kellmann et al., 2018). Items were framed to capture athletes’ perceived psychological restoration following recent training sessions rather than general wellbeing. Specifically, items reflect detachment, emotional restoration, and regained psychological energy following exertion.

## 6 Analytical strategy

To test the proposed conditional process model, we employed partial least squares structural equation modeling (PLS-SEM) using SmartPLS 4.0. PLS-SEM was selected due to its suitability for complex models incorporating mediation and moderation effects, its robustness with latent constructs measured by multiple indicators, and its emphasis on variance explanation and prediction (Hair et al., 2019; Hair et al., 2021).

The analytic procedure followed a structured sequence. First, the measurement model was evaluated to assess indicator reliability, internal consistency reliability, convergent validity, and discriminant validity. Second, the structural model was examined by evaluating collinearity diagnostics, path coefficients, coefficient of determination (R^2^), and effect sizes. Third, mediation effects were tested using bootstrapping procedures with 5,000 resamples to estimate indirect effects and confidence intervals. Fourth, moderation effects were assessed by creating interaction terms between self-regulation and perceived control, and their significance was evaluated through bootstrapped confidence intervals. Finally, the conditional indirect (moderated mediation) effects were examined following established procedures for variance-based structural modeling (Hayes, 2018), with conditional effects probed at different levels of the moderator. This stepwise analytic plan ensured a systematic evaluation of both measurement quality and hypothesized structural relationships.

## 7 Results

The study utilizes partial least squares structural equation modeling (PLS-SEM) to test the proposed moderated mediation framework. PLS-SEM was selected because it is particularly suitable for models that are prediction-oriented and structurally complex, involving multiple latent constructs, mediating mechanisms, moderating effects, and conditional indirect relationships. Unlike covariance-based SEM, PLS-SEM does not impose strict distributional assumptions and performs robustly when estimating interaction terms and indirect effects simultaneously, making it especially appropriate for theory development and exploratory extensions of existing frameworks. Moreover, PLS-SEM is widely recommended when the primary objective is to explain variance in key endogenous constructs and assess predictive relevance rather than overall model fit alone (Hair et al., 2021).

Table 1 presents the measurement model results, including indicator reliability, internal consistency reliability, and convergent validity. All constructs demonstrate satisfactory reliability, with Cronbach’s alpha and composite reliability values exceeding the recommended threshold of 0.70, indicating adequate internal consistency. In addition, average variance extracted (AVE) values for all latent variables exceed 0.50, confirming that each construct explains more than half of the variance in its indicators. These results provide strong support for convergent validity and suggest that the measurement items adequately capture their respective theoretical constructs. Collectively, the findings reported in Table 1 indicate that the reflective measurement model meets established psychometric quality criteria and is suitable for subsequent structural model evaluation (Fornell and Larcker, 1981; Hair et al., 2021).

**Table 1:** Measurement model assessment.

Latent variables	Outer loadings	Cronbach’s alpha	Composite reliability	Average variance extracted
Cognitive flexibility		0.878	0.908	0.621
CF1	0.795			
CF2	0.803			
CF3	0.785			
CF4	0.791			
CF5	0.803			
CF6	0.750			
Perceived control		0.927	0.945	0.773
PC1	0.883			
PC2	0.885			
PC3	0.899			
PC4	0.871			
PC5	0.858			
Psychological recovery		0.876	0.906	0.617
PR1	0.758			
PR2	0.779			
PR3	0.801			
PR4	0.806			
PR5	0.769			
PR6	0.799			
Self-regulation		0.916	0.931	0.629
SR1	0.794			
SR2	0.768			
SR3	0.783			
SR4	0.801			
SR5	0.805			
SR6	0.837			
SR7	0.796			
SR8	0.757			

Discriminant validity was further examined using the Fornell–Larcker and the heterotrait–monotrait ratio of correlations (HTMT), as reported in Table 2. As reported in Table 2, the square root of the AVE for each construct is greater than its corresponding inter-construct correlations, indicating that each latent variable shares more variance with its associated indicators than with other constructs in the model. In addition, the HTMT analysis shows that All HTMT values fall well below the conservative cutoff value of 0.85, indicating that the constructs are empirically distinct from one another. The HTMT criterion has been shown to be more sensitive than traditional approaches such as the Fornell–Larcker criterion in detecting discriminant validity issues, particularly in variance-based SEM (Henseler et al., 2015). The results therefore provide robust evidence that the latent variables represent conceptually and empirically separable constructs, allowing meaningful interpretation of the structural relationships among them (Henseler et al., 2015; Hair et al., 2021).

**Table 2:** Discriminant validity.

Latent variables	1	2	3	4
Fornell–Larcker				
1. Cognitive flexibility	0.788			
2. Perceived control	0.132	0.879		
3. Psychological recovery	0.447	0.226	0.786	
4. Self-regulation	0.454	0.227	0.522	0.793
Heterotrait–monotrait ratio				
1. Cognitive flexibility				
2. Perceived control	0.144			
3. Psychological recovery	0.506	0.247		
4. Self-regulation	0.502	0.242	0.579	

Table 3 presents the global model fit indices for the saturated and estimated models. The standardized root mean square residual (SRMR) value of 0.053 for both models is below the recommended threshold of 0.08, indicating an acceptable approximate model fit. Similarly, the d_ULS and d_G values are low and show negligible differences between the saturated and estimated models, suggesting that the specified structural relationships do not meaningfully distort the observed correlation matrix. The chi-square values are reported for descriptive purposes, as chi-square is highly sensitive to sample size and is not used as a strict fit criterion in PLS-SEM. The normed fit index (NFI) values of 0.856 and 0.857 exceed the commonly accepted minimum of 0.80, indicating an adequate incremental fit relative to the null model. Overall, these indices collectively support the adequacy of the proposed model and justify proceeding with interpretation of the structural relationships (Hair et al., 2019; Hair et al., 2021).

**Table 3:** Model fit.

Fit metrics	Saturated model	Estimated model
SRMR	0.053	0.053
d_ULS	0.925	0.919
d_G	0.392	0.391
Chi-Square	1,071.839	1,069.298
NFI	0.856	0.857

Table 4 reports the structural model results and hypothesis testing based on bootstrapping procedures. The findings indicate that cognitive flexibility is significantly positively associated with self-regulation (*β* = 0.381, *p* < 0.001), indicating that athletes reporting higher cognitively flexible also report stronger regulate their thoughts, emotions, and behaviors. Self-regulation, in turn, is significantly and positively associated with psychological recovery (*β* = 0.366, *p* < 0.001), supporting the view that effective regulatory capacities facilitate athletes’ recovery from competitive stress. The indirect effect of cognitive flexibility on psychological recovery via self-regulation is also significant (*β* = 0.139, *p* < 0.001), supporting the proposed mediating association of self-regulation and indicating that the association between cognitive flexibility and recovery is statistically transmitted through self-regulatory processes.

**Table 4:** Structural equation model.

Hypotheses	*B*	CIs	S.E.	*t*-value	*p*-value
Cognitive flexibility → Self-regulation	0.381	0.262, 0.473	0.055	6.904	0.000
Self-regulation → Psychological recovery	0.366	0.261, 0.487	0.057	6.403	0.000
Cognitive flexibility → Self-regulation → Psychological recovery	0.139	0.083, 0.211	0.033	4.285	0.000
Moderating effect → Self-regulation	−0.149	−0.262, −0.228	0.061	2.445	0.015
Moderated mediating effect → Psychological recovery	−0.075	−0.176, −0.023	0.028	2.678	0.010

In addition, the moderating effect on the self-regulation pathway is significant and negative (*β* = −0.149, *p* = 0.015), indicating that the positive relationship between self-regulation and psychological recovery becomes weaker as perceived control increases. Although psychological recovery remains higher overall among athletes reporting greater perceived control, the incremental effect of self-regulation on recovery is comparatively stronger under lower perceived control and attenuated under higher perceived control. In other words, self-regulatory capacity shows a stronger association with recovery when athletes perceive limited control over their performance context, whereas its marginal contribution diminishes when perceived control is already high.

Moreover, the moderated mediation effect on psychological recovery is statistically significant (*β* = −0.075, *p* = 0.010), providing evidence that the indirect relationship between cognitive flexibility and psychological recovery through self-regulation is conditional on the level of the moderator. Specifically, the indirect effect is stronger at lower levels of perceived control and weaker at higher levels of perceived control, reflecting a conditional process in which contextual appraisals shape the strength of the resource-based recovery pathway.

**Figure 2:**
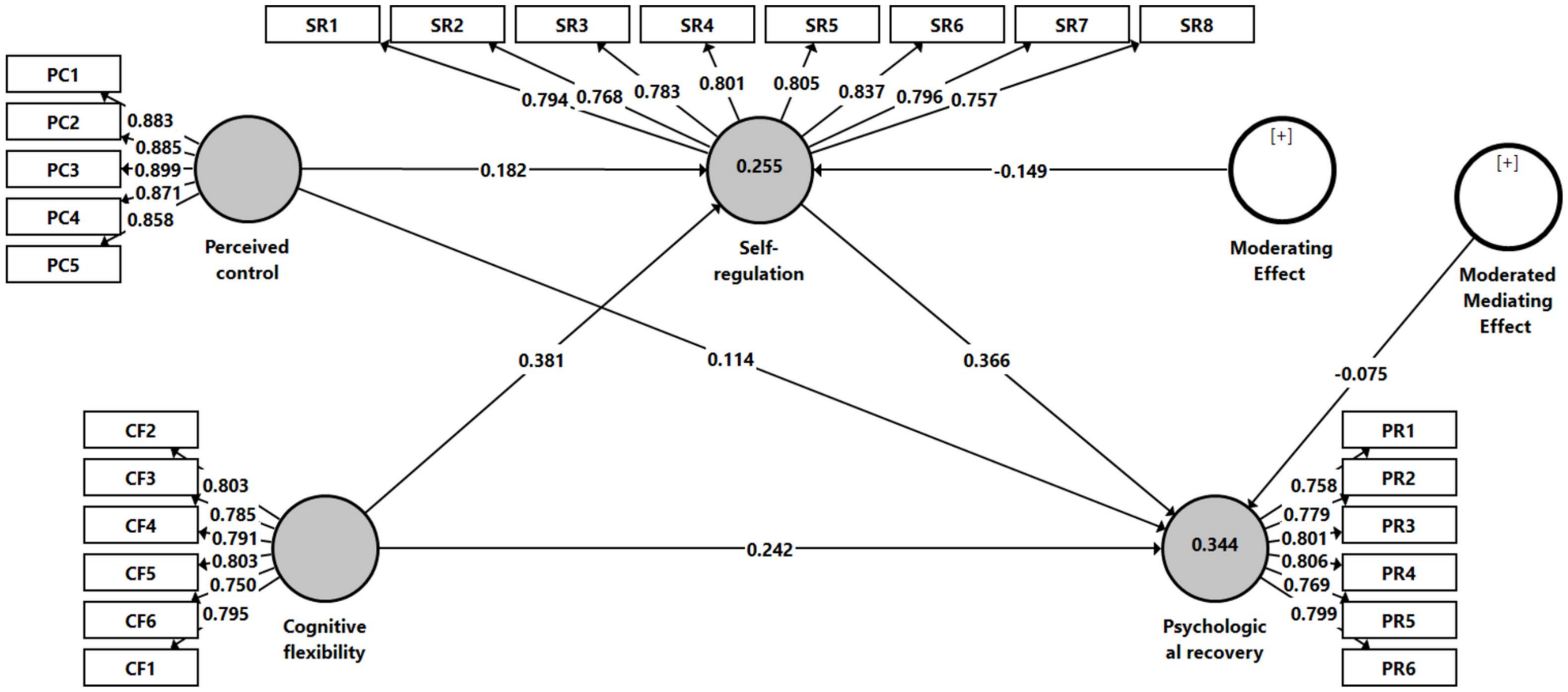
Structural equation model.

**Figure 3:**
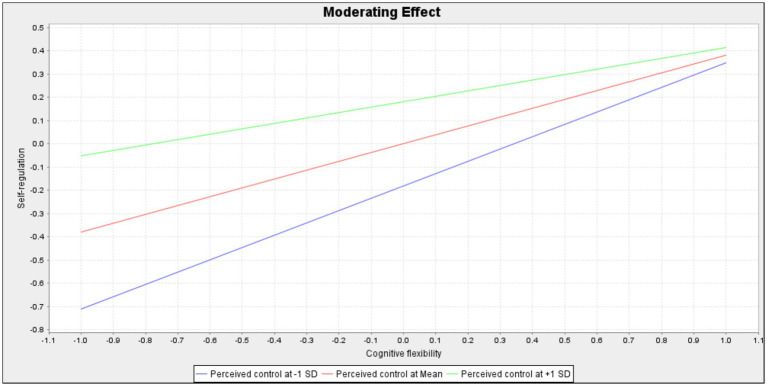
Moderating effect.

**Figure 4:**
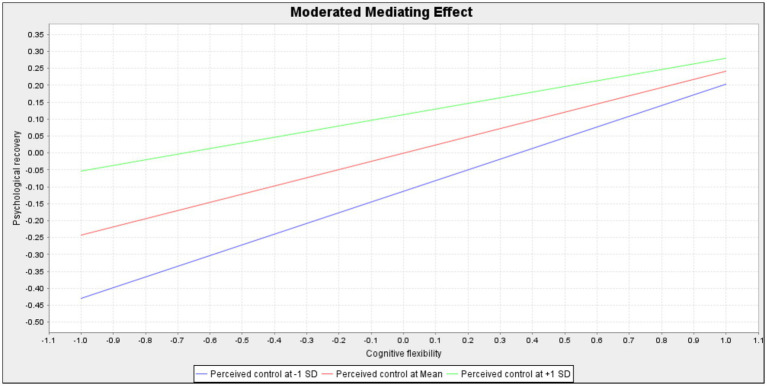
Moderated mediating effect.

These structural relationships are visually depicted in Figure 2, while Figures 3 and 4 illustrate the conditional slopes and indirect effects, respectively, thereby reinforcing the robustness and coherence of the proposed conditional process model (Hair et al., 2019; Hair et al., 2021; Hayes, 2018). Figure 3 illustrates that the slope of self-regulation predicting psychological recovery is steeper under low perceived control and flatter under high perceived control. Figure 4 further demonstrates that the conditional indirect effect of cognitive flexibility through self-regulation is stronger at lower levels of perceived control and attenuated at higher levels.

Table 5 presents the results of the blindfolding procedure used to assess the predictive relevance of the structural model using the Stone–Geisser *Q*^2^ criterion with an omission distance of five. As shown in the table, positive *Q*^2^ values are obtained for the endogenous constructs psychological recovery (*Q*^2^ = 0.199) and self-regulation (*Q*^2^ = 0.147), indicating that the model exhibits satisfactory predictive relevance for these key outcome variables. According to established guidelines, *Q*^2^ values greater than zero suggest that the exogenous constructs have meaningful out-of-sample predictive power for the endogenous variables, thereby extending support for the model beyond explanatory adequacy (Stone, 1974; Geisser, 1974; Hair et al., 2019; Hair et al., 2021).

**Table 5:** Blindfolding.

Latent variables	SSO	SSE	*Q*^2^ (=1-SSE/SSO)
Cognitive flexibility	2,802.000	2,802.000	
Moderated mediating effect	467.000	467.000	
Moderating effect	467.000	467.000	
Perceived control	2,335.000	2,335.000	
Psychological recovery	2,802.000	2,244.716	0.199
Self-regulation	3,736.000	3,186.449	0.147

## 8 Discussion

The findings offer convergent support for the proposed conditional process model by showing that cognitive flexibility is positively associated with psychological recovery partly through self-regulation, while the magnitude of this indirect pathway depends on the moderator. The significant positive association between cognitive flexibility and self-regulation is consistent with prior sport and performance research indicating that athletes who can shift perspectives, adapt to situational demands, and reframe setbacks tend to demonstrate stronger regulatory functioning, including better planning, attentional control, and emotion management under pressure. In athletic contexts, cognitive flexibility has been linked with better coping and lower stress-related responses, particularly among higher-performing athletes, suggesting that flexible cognitive processing helps athletes respond more effectively to competitive demands and maintain functional control over behavior during performance (Kim et al., 2025). This pattern is also theoretically coherent with the broader view that adaptive cognition provides the psychological readiness needed to initiate and sustain goal-directed self-regulation in dynamic environments such as competition (Arı and Korkmaz, 2025).

The direct positive effect of self-regulation on psychological recovery aligns closely with scholarship emphasizing that recovery is not merely a passive outcome of rest but an active, self-managed process that requires intentional regulation of thoughts, emotions, and behaviors after training and competition. Prior work in sport psychology shows that athletes with stronger self-regulatory skills display more effective coping, superior performance-related learning, and greater capacity to manage strain, which together facilitate restoration and reduce cumulative stress. For example, self-regulation has been associated with higher performance levels in youth athletes, reflecting that systematic self-management supports sustained functioning and adaptive adjustment across demanding seasons (Sakalidis et al., 2022). More directly tied to recovery processes, conceptual and empirical work on recovery self-regulation suggests that athletes who can deliberately downregulate arousal, structure recovery behaviors, and manage cognitive-emotional spillover are more likely to regain resources following stress exposure (Wilson and Young, 2024). These results also resonate with recovery research emphasizing that adequate recovery is essential for maintaining high performance and preventing maladaptive outcomes, including overtraining and burnout, particularly in high-demand competitive sport environments (Kellmann et al., 2024; Tang et al., 2022). Complementing this perspective, evidence from health-related sport samples suggests that lower recovery and higher stress states can foreshadow mental health problems, reinforcing the practical relevance of recovery as a psychological outcome and the importance of mechanisms that strengthen it, such as self-regulatory capacity (Bakker and De Vries, 2021; Pozzato et al., 2024).

The significant indirect effect indicates that self-regulation partially transmits the beneficial influence of cognitive flexibility to psychological recovery, which supports a resource-based interpretation of post-competition adaptation. Conservation of resources theory proposes that individuals cope more effectively with stress when they possess and can mobilize valued resources, and that resources tend to accumulate in gain spirals, whereas depletion can initiate loss spirals (Hobfoll, 1989). In this framing, cognitive flexibility can be understood as a personal resource that enables athletes to appraise demands adaptively and avoid rigid responses, thereby supporting self-regulatory investment. Self-regulation, in turn, functions as a higher-order resource enabling athletes to implement recovery behaviors, re-establish equilibrium, and restore capacity after exertion. The present mediation finding therefore provides empirical support for the proposition that flexible cognition may be associated with recovery through its relationship with athletes’ regulatory processes (Balk and Englert, 2020).

Importantly, the significant moderating effect and the significant moderated mediation effect indicate that the mediated pathway operates differently across levels of the moderator, suggesting that the translation of self-regulatory capacity into recovery outcomes is contingent on athletes’ contextual or psychological conditions. This pattern is consistent with research highlighting that perceived control and goal-related appraisals can shape recovery and stress responses in athletes, particularly during critical periods such as tapering and pre-competition phases (Bostan et al., 2022; Forsblom et al., 2022). Similarly, studies in swimming contexts have suggested that perceived control is associated with more favorable recovery and stress states, indicating that athletes’ appraisals of control can amplify or dampen the effectiveness of coping and recovery efforts (Alvarez et al., 2021; Zhu et al., 2025).

More specifically within sport training contexts, the observed pattern indicates that when athletes perceive lower control over training intensity, selection decisions, or competitive uncertainty, self-regulation becomes a compensatory internal mechanism that plays a more decisive role in facilitating psychological recovery. Under conditions of limited perceived control—such as externally imposed tactical roles, unexpected lineup changes, or rigid coaching structures—athletes may rely more heavily on deliberate attentional shifting, emotional downregulation, and structured recovery planning to restore depleted resources. In contrast, when perceived control is already high, athletes may experience elevated baseline confidence and agency, reducing the marginal contribution of additional self-regulatory effort to recovery outcomes. In some cases, excessively high perceived control may even foster overinvestment in self-management or excessive cognitive monitoring, potentially leading to overexertion in regulation and diminished recovery efficiency.

This compensatory and potentially diminishing-return dynamic aligns with conservation of resources theory (Hobfoll, 1989), which suggests that personal resources exert stronger effects when contextual resources are constrained, whereas the incremental value of additional resource investment decreases when contextual support is already abundant.

Theoretical implications.

The present study makes several important theoretical contributions by extending and integrating distinct strands of literature on cognitive processes, self-regulatory mechanisms, and recovery in competitive sport. First, the study advances recovery research by positioning psychological recovery not merely as a passive outcome of rest or reduced load but as an outcome of active, cognitively driven self-regulatory processes. While prior studies have acknowledged the role of self-regulation in performance and coping, they have largely treated recovery as a downstream consequence without explicitly modeling its cognitive antecedents (Fogaca, 2021; Hagger and Orbell, 2022). By introducing cognitive flexibility as an upstream cognitive resource, the study addresses a notable gap in the literature where adaptive cognition and recovery processes have remained theoretically disconnected. In doing so, the study reframes psychological recovery as a resource mobilization process consistent with conservation of resources (COR) theory (Hobfoll, 1989; Hobfoll et al., 2018), wherein adaptive cognitive capacity represents an upstream personal resource associated with downstream regulatory investment and restoration. This perspective extends recovery self-regulation frameworks in sport (Balk and Englert, 2020) by linking cognitive flexibility to the mobilization of recovery-oriented regulatory strategies.

Second, the study extends self-regulation theory in sport by empirically demonstrating that self-regulation functions as a key explanatory mechanism linking higher-order cognitive capacities to recovery-related outcomes, thereby clarifying how abstract cognitive traits are translated into tangible psychological restoration. Rather than conceptualizing self-regulation as a static dispositional attribute, the present findings suggest that it operates as a dynamic regulatory process through which cognitive flexibility is expressed in recovery-relevant behaviors, such as attentional disengagement and emotional modulation (Balk and Englert, 2020; Wilson and Young, 2024). This process-oriented clarification aligns with contemporary sport psychology models emphasizing active recovery management rather than passive rest (Eccles et al., 2022).

Third, the inclusion of perceived control as a conditioning variable contributes to theory by showing that the effectiveness of self-regulatory processes is not uniform but contingent on athletes’ control appraisals, highlighting the importance of contextual and appraisal-based boundary conditions. Importantly, the observed compensatory pattern—whereby the association between self-regulation and psychological recovery is stronger under lower perceived control and attenuated under higher perceived control—refines existing resource-based assumptions that contextual assets uniformly amplify personal resource effects. Instead, the findings are consistent with COR principles suggesting that personal resources may exert stronger effects when contextual resources are constrained and diminishing marginal effects when contextual support is already abundant (Hobfoll, 1989; Hobfoll et al., 2018). This nuanced interpretation advances theoretical understanding of how personal and contextual resources interact in competitive sport environments.

This conditional process perspective responds directly to calls in the literature for more nuanced models that move beyond simple direct or mediated effects and instead capture when and for whom psychological mechanisms operate most effectively (Hayes, 2018). More broadly, the moderated mediation framework integrates cognitive, motivational, and recovery-based perspectives into a single explanatory model, thereby expanding existing theories that have traditionally examined these constructs in isolation. By explicitly modeling the interplay between cognitive resources, regulatory processes, and contextual appraisals, the study contributes to a more ecologically grounded understanding of recovery as a context-sensitive and resource-dependent process (Balk and Englert, 2020; Eccles et al., 2022). In doing so, the study offers a refined conceptual foundation for future theory building in sport psychology and related performance domains.

### 8.1 Practical implications

The findings of this study offer several practical implications for coaches, sport psychologists, and athletic organizations seeking to promote sustainable performance and wellbeing among competitive athletes. First, the results suggest that psychological recovery may be positively associated with higher levels of cognitive flexibility, as flexible thinking appears to be positively associated with more effective self-regulation and recovery-related experiences. Practitioners may integrate cognitive flexibility training into regular psychological skills programs through techniques such as cognitive reframing, structured post-match reflection sessions, video-assisted feedback analysis in which athletes reinterpret performance errors from multiple perspectives, and situational simulation drills that expose athletes to unexpected tactical changes requiring rapid cognitive adjustment. For example, coaches may design training sessions in which athletes are required to adapt to sudden rule modifications or altered performance constraints, thereby strengthening adaptive appraisal processes that later support recovery from competitive stress.

Second, the central role of self-regulation highlights the importance of equipping athletes with concrete regulatory skills, including structured goal-setting protocols, guided breathing and arousal-regulation routines following high-intensity training blocks, attentional refocusing scripts, and individualized recovery planning templates. Practical interventions may include implementing post-training “regulation windows” in which athletes engage in guided detachment exercises, journaling of cognitive-emotional states, or coach-supervised recovery planning meetings to prevent rumination and cognitive spillover into subsequent sessions.

Third, the conditional role of perceived control underscores the importance of calibrating control experiences within sport environments. Rather than uniformly increasing perceived control, practitioners may aim to optimize adaptive control perceptions while avoiding excessive overconfidence or hyper-control tendencies that encourage overexertion in self-management. Coaches can operationalize this by involving athletes in structured decision-making discussions, allowing controlled autonomy in recovery choices (e.g., selection of recovery modality), and providing feedback that emphasizes controllable process factors rather than uncontrollable outcomes.

Collectively, these implications suggest that effective recovery interventions should adopt an integrated approach that simultaneously targets cognitive adaptability, self-regulatory competence, and calibrated control appraisals, thereby helping athletes maintain psychological resilience and sustain high-level performance over time.

### 8.2 Limitations and future research directions

Despite its contributions, the present study has several limitations that should be acknowledged and addressed in future research. First, the cross-sectional research design limits the ability to draw causal inferences about the relationships among cognitive flexibility, self-regulation, perceived control, and psychological recovery. Although the proposed model is theoretically grounded, the observed associations reflect statistical relationships at a single time point and do not establish temporal ordering or directional causality. Given that recovery processes in sport are inherently dynamic and fluctuate across training microcycles, competitive phases, and tapering periods, longitudinal, diary-based, or time-lagged designs would be better suited to capture within-athlete variability and reciprocal influences among these constructs across time.

Second, the reliance on self-reported data may introduce common method bias and social desirability effects, as athletes’ perceptions of their cognitive and regulatory capacities may not fully align with objective behaviors or physiological recovery indicators. In addition, psychological recovery was assessed as a perceived state, which may be influenced by transient contextual factors such as recent performance outcomes, acute fatigue, or interpersonal feedback. Future studies may benefit from combining subjective recovery reports with physiological indices (e.g., heart rate variability, cortisol measures), behavioral tracking of recovery routines, or coach evaluations to strengthen construct validity and reduce mono-method bias.

Third, the sample was drawn exclusively from competitive athletes in China, which may limit the generalizability of the findings to other cultural or sporting contexts. Given that perceived control and regulatory practices may be shaped by coaching norms, hierarchical structures, and cultural attitudes toward authority and autonomy, cross-cultural replications are particularly important for determining the boundary conditions of the observed moderation effect. Comparative research across individual versus team sports, or across developmental versus elite levels, would further clarify contextual variability.

Fourth, although PLS-SEM was selected to model complex conditional pathways and latent constructs, the present analysis remains correlational in nature. Future research may complement variance-based modeling approaches with experimental or quasi-experimental designs to test whether deliberate manipulation of cognitive flexibility training, self-regulatory skill development, or perceived control interventions produces measurable changes in recovery outcomes.

In addition, future research may extend the present framework by examining additional contextual moderators, such as coaching style, team climate, competitive level, or training load variability, to further refine understanding of when self-regulatory mechanisms most effectively translate into recovery experiences. Finally, intervention-based studies are encouraged to examine whether structured cognitive flexibility programs, recovery self-regulation workshops, or autonomy-supportive coaching practices are associated with sustained improvements in psychological recovery and long-term athlete wellbeing across competitive seasons.

## 9 Conclusion

This study provides a comprehensive examination of the psychological mechanisms underlying recovery among competitive athletes by integrating cognitive flexibility, self-regulation, and perceived control within a moderated mediation framework. The findings accentuate the importance of viewing psychological recovery as an active, resource-dependent process shaped by athletes’ cognitive capacities and regulatory skills rather than as a passive consequence of rest alone. By demonstrating that cognitive flexibility is associated with psychological recovery both directly and indirectly through self-regulation, and that this indirect association is contingent on perceived control, the study advances theoretical understanding of how adaptive cognition and self-management jointly support recovery in demanding performance contexts. Collectively, the results highlight the value of adopting an integrative perspective that accounts for cognitive, regulatory, and contextual factors in sustaining athletes’ psychological wellbeing. The proposed framework offers a solid foundation for future research and practice aimed at promoting sustainable performance and long-term resilience in competitive sport.

## Data Availability

The original contributions presented in the study are included in the article/supplementary material, further inquiries can be directed to the corresponding author.

## Appendix A: Measurement items

### Cognitive flexibility

- CF1: During the past week, I was able to change my thinking when situations in competition changed.
- CF2: When faced with a difficult training situation, I considered multiple ways to handle it.
- CF3: I could easily adjust my strategies when my initial plan did not work.
- CF4: I was open to different perspectives about my performance.
- CF5: I was able to see challenging situations from alternative viewpoints.
- CF6: I quickly adapted my thinking when unexpected events occurred.

### Self-regulation

- SR1: During the past week, I set clear goals for my training and competition.
- SR2: I monitored my progress toward my performance goals.
- SR3: I was able to control my emotions during demanding situations.
- SR4: I stayed focused on my tasks despite distractions.
- SR5: I adjusted my effort when I noticed that I was not performing optimally.
- SR6: I evaluated my performance to improve future outcomes.
- SR7: I managed my time effectively for recovery and preparation.
- SR8: I maintained discipline even when I felt fatigued.

### Perceived control

- PC1: During the past week, I felt that I could influence my performance outcomes.
- PC2: I believed that my actions made a difference in competition results.
- PC3: I felt in control of how I responded to challenges.
- PC4: I was confident that I could manage unexpected difficulties.
- PC5: I felt that I had control over my training progress.

### Psychological recovery

- PR1: During the past week, I was able to mentally detach from sport after training or competition.
- PR2: I felt mentally refreshed after resting.
- PR3: I recovered emotionally after demanding sessions.
- PR4: I felt restored before the next training session.
- PR5: I was able to relax after competition.
- PR6: I regained psychological energy following exertion.
